# Effect of superfine grinding on physicochemical and antioxidant properties of soybean residue powder

**DOI:** 10.1002/fsn3.1409

**Published:** 2020-01-27

**Authors:** Guanghui Li, Weiyun Guo, Xueli Gao, Yonghui Wang, Sisheng Sun

**Affiliations:** ^1^ Food and Bioengineering College Xuchang University Xuchang Henan China; ^2^ Key Laboratory of Biomarker Based Rapid‐detection Technology for Food Safety of Henan Province Xuchang Henan China

**Keywords:** antioxidant activity, physicochemical properties, soybean residue, superfine grinding

## Abstract

Soybean residue is an underutilized, nutrient‐rich by‐product of soybean processing. To enhance its value, we subjected soybean residue to superfine grinding and measured the resulting physiochemical properties and antioxidant activities. We prepared powders with particle sizes of 115.35, 77.93, 39.38, 25.01, and 20.44 μm. As particle size decreased, the surface area (from 96.46 to 198.32 m^2^/kg) and swelling capacity (from 2.05 to 10.62 ml/g) increased. Conversely, we observed decreases in the surface‐number mean (from 23.07 to 11.20 μm), volume‐surface mean (from 141.70 to 27.96 μm), angles of repose (from 48.30° to 31.46°), water holding capacity (from 7.86 to 4.39 g/g), and oil binding capacity (from 1.78 to 1.42 g/g). The water solubility index and antioxidant activity (reducing power and free radical scavenging activities of 2,2‐diphenyl‐1‐picrylhydrazyl and 2,2′‐azino‐di‐(3‐ethylbenzthiazoline sulfonic acid)) improved as particle size decreased. In conclusion, superfine grinding improved some properties of soybean residue. Additionally, our findings provide theoretical support for using superfine grinding in industrial food applications.

## Graphical abstract

Superfine grinding improved the physicochemical and functional properties of soybean residue. Antioxidant activity could be improved by the superfine grinding.

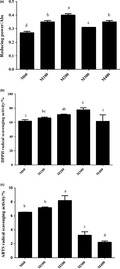

## 1 INTRODUCTION

Soybean products, such as soy milk, tofu, yuba, and tofu skin, are the most popular foods worldwide (Chen et al., 2012; Thakur, Wei, & Tomar, 2020). However, soybean residue is generated during processing (Li et al., 2013). In China, a large quantity, >2.8 million tons, of soybean residue is obtained just from the tofu industry each year (Li, Qiao, & Lu, 2012). Soybean residue contains protein, carbohydrates, dietary fiber, isoflavone, and minerals (Li et al., 2013; O'Toole, 1999). It has antioxidant activity and may aid in prevention of diabetes, obesity, and hyperlipidemia (Li et al., 2012; Li et al., 2017; Li et al., 2014; Wang, Zhou, Hou, Qi, & Zhang, 2013). However, soybean residue is not widely used in food because of its high moisture content and poor edible quality. A greater concern is the fact that most soybean residues are dumped, causing environmental problems. Attempts have been made to use soybean residue as animal feed, fertilizer, and food ingredients (Li et al., 2012).

Superfine grinding is a useful tool for obtaining powders with high solubility, dispersion, adsorption, and fluidity (Zhang, Zhang, & Shrestha, 2005). Superfine grinding technology has been applied in biotechnology, the pharmaceutical industry, and food processing (Zhao et al., 2009; Zhong et al., 2016). Superfine powders from tea, qingke, Lycium barbarum, and wheat flour have been produced by different ultrafine grinding techniques (Hu, Chen, & Ni, 2012; Niu, Hou, Wang, & Chen, 2014; Zhang et al., 2014; Zhu, Du, & Xu, 2015).

Although the chemical composition, usage, and health benefits of soybean residue were examined in several studies (Li et al., 2012; Li et al., 2013), there is little information about the effect of the superfine grinding technology on the properties of soybean residue. Therefore, the aim of this study was to evaluate the properties of soybean residue subjected to superfine grinding. We measured particle size, color, angle of repose, water holding capacity, oil binding capacity, swelling capacity, water solubility index, and antioxidant properties. And improved properties of soybean residue powder were found, which were made by superfine grinding.

## 2 MATERIALS AND METHODS

### 2.1 Materials

Soybean residue was obtained from the bean curd workshops in Xuchang City, Henan Province, China. DPPH (2,2‐diphenyl‐1‐picrylhydrazyl) and ABTS (2,2′‐azino‐di‐(3‐ethylbenzthiazoline sulfonic acid) were purchased from Sigma Chemical Company. Ethyl alcohol was provided by Fuyu Chemical Company. Pyrogallol, methanol, FeSO_4_, K_2_S_2_O_8,_ and FeCl_3_ were purchased from Kemio Chemical Company. All chemicals and reagents were analytical grade.

### 2.2 Preparation of Soybean residue powders

Soybean residue was dried in an air oven (45°C) to reduce the water content below 5% (w/w). The dried residue was milled by a high speed multi‐function pulverizer (2500A; Sufeng Industry & Trade Co., Ltd.) for 2 min. The powder was further milled in an NLD‐6DI type micronizer (Nalide Superfine Grinding Technology Co., Ltd.) for 20 min and passed through screens with mesh size of 60, 100, 200, 300, and 400; the powders were named as M60, M100, M200, M300, and M400.

### 2.3 Particle size analysis

The particle size distribution of soybean residue was determined with a laser particle size analyzer (BT‐9300S; Bettersize Instruments Ltd.).

### 2.4 Color difference analysis

The color of soybean residue powder was measured using the NR200 portable colorimeter (3nh Scientific Co., Ltd.).

### 2.5 Determination of the angle of repose

The angle of repose was measured as described by Zhong et al. (2016). First, a funnel was fixed vertically above graph paper; the distance (H) between the paper and the outlet of the funnel was 3 cm. Then, the powder was poured into the funnel until the tip of the powder cone just touched the outlet of the funnel. The diameter (R) of the cone was determined. The angle of repose (*θ*) was calculated by the following relationship:

$$\theta =\text{arctg}\left(\text{R/H}\right)$$

### 2.6 Determination of SC

Swelling capacity (SC) was measured according to the method of Lecumberri et al. (2007). First, 1 g of sample (M) was weighed accurately, then the powder was put into a calibrated tube, and the bed volume (*V*_1_) was recorded. Distilled water (10 ml) was added to the calibrated tube and mixed with the powder. After shaking well, the tube was kept in a water bath (25°C) for 24 hr. The bed volume (*V*_2_) was recorded again. SC was calculated using the following equation:

$$\text{SC}\left(\text{ml/g}\right)=\left(V_{2}-V_{1}\right)/M$$

### 2.7 Determination of WHC and OBC

Water holding capacity (WHC) was measured by the method of Zhang et al. (2005), with minor modification. In brief, a centrifuge tube (M) was accurately weighted. One g of sample (M_1_) and 10 ml of distilled water (M_2_) were poured into the tube and mixed uniformly by a vortex. The tube was kept in a water bath (60°C, 30 min) and then centrifuged (15 min, 4,000 rpm). The supernatant was removed, and the centrifuge tube with the wet powder (M_3_) was weighed again. The WHC was calculated using the following equation:

$$\text{WBC}\;\left(\text{g/g}\right)=\;\left(M_{3}-M-M_{1}\right)/M_{1}$$

The oil binding capacity (OBC) of the soybean residue powder was determined by the method of Sangnark and Noomhorm with a modification (2003). The centrifuge tube (*m*_1_) was weighed. Two gram of the powder (*m*_0_) and 10 ml of soybean oil were added to the tube. The tube was kept in a water bath (25°C) for 1 hr, then the mixture was centrifuged (15,000 *g*, 10 min). The supernatant was discarded, and the weight of the tube with the wet powder (*m*_2_) was measured again. The OBC was calculated by the following equation:

$$\text{OBC}\;\;\left(\text{g/g}\right)\;=\;\left(m_{2}-m_{1}-m_{0}\right)/m_{0}$$

### 2.8 WSI assay

The water solubility index (WSI) was measured as reported by Zhao, Ao, et al. (2009) with some slight modifications. Briefly, 1.0 g of sample was weighed accurately and mixed with distilled water (50 ml) in a tube. The tube was kept in a water bath (37°C) and shaken for 30 min. Then, the tube was centrifuged (7,500 *g*, 10 min), and the supernatant was discarded. Finally, the sample was dried in an oven (100°C). After 12 hr, the weight of sample (*A*) was determined again. The WSI was calculated with the following relationship:

$$\text{WSI}\;\left(\%\right)\;=\;A\times 100\%$$

### 2.9 Preparation of extracts

Samples (5 g) were extracted in 100 ml of 70% ethanol in an ultrasonic cleaner (Tianhuachaosheng Instrument Company) at 45°C, 320 W for 30 min. The extract was filtered, and the residue was extracted again with the same conditions. The filtrates were combined and used for the following assays.

### 2.10 Determination of reducing power

The reducing power of the extracts was determined by the method of Lim and Murtijaya (2007) with some modifications. In brief, 2.5 ml of extract was mixed with 2.5 ml of sodium phosphate buffer (pH 6.6, 0.2 mol/L) and 2.5 ml of 1% potassium ferricyanide; the mixture was incubated in a water bath (50°C) for 20 min. Trichloroacetic acid (2.5 ml of 10%) was added to the mixture, followed by 5.0 ml distilled water and 1.0 ml of 0.1% ferric chloride. After 30 min, optical density (OD) at 700 nm was measured with a UV‐7504 spectrophotometer (Shanghai Five Phase Instrument Co., Ltd.). An increase in absorbance corresponded to an increase in reducing power.

### 2.11 Determination of DPPH radical scavenging capacity

DPPH radical scavenging capacity was determined by a modification of the method of Gyamfi, Yonamine, and Aniya (1999). Briefly, 4 ml of extract was mixed with 1 ml of 0.2 mM DPPH (in methanol). The mixture was shaken vigorously and kept in the dark for 30 min. The OD_517nm_ was measured by a spectrophotometer. DPPH scavenging ability was calculated by the following equation:

$$\text{Scavenging ability}\;\left(\%\right)\;=\;\left[1-\left(A_{X}-A_{X0}\right)/A_{0}\right)]\times 100$$

where *A*_x_ is the absorbance of the sample plus DPPH; *A*_x0_ is the absorbance of the sample; and *A*_0_ is the absorbance of distilled water plus DPPH.

### 2.12 Determination of ABTS radical scavenging capacity

ABTS scavenging capacity of the powders was assessed according to Fan et al. (2009) with minor modifications. ABTS radical cation was obtained by reacting 7 mM ABTS with 2.45 mM potassium persulphate, in the dark for 12 hr at 25°C. The ABTS solution was diluted with PBS (pH 7.0) to an absorbance of 0.70 ± 0.02 at 734 nm before using. Extracts (0.5 ml) were mixed uniformly with 3 ml of ABTS solution. The mixture was incubated at 25°C for 6 min. The OD_734nm_ was measured with a spectrophotometer. The ABTS scavenging effect was calculated as follows:

$$\text{ABTS scavenging activity}\;\left(\%\right)\;=\;\left[1-\left(A-A_{b}\right)/A_{0}\right)]\;\times \;100.$$

where *A*_0_ is the absorbance of ABTS solution without sample; *A* is the absorbance of the sample plus ABTS solution; and *A*_b_ is the absorbance of the sample without ABTS solution.

### 2.13 Statistical analysis

All measurements were performed three times. The values were means ± standard deviations (*SD*). The Design of Experiment‐Processing of Data‐Statistical Analysis (DPS, Version 6.55, Refine Information Tech. Co., Ltd.) was used to analyze the data differences. A value of *p* < .05 was considered statistically significant.

## 3 RESULTS AND DISCUSSION

### 3.1 Particle size distribution

Table 1 shows the particle size distribution of the five soybean residue powders. Generally, we used D_10_, D_50,_ and D_90_ to characterize particle size distributions. D_10_, D_50,_ and D_90_ are the equivalent volume diameters at 10%, 50%, and 90% cumulative volume, respectively. As the particle size of soybean residue powder decreased, D_10_, D_50,_ and D_90_ decreased significantly, from 11.29 to 5.26 μm, 115.35 to 20.44 μm, and 311.33 to 60.17 μm, respectively. D_50_, represented the degree of powder cohesiveness, is the average median diameter, and the D_50_ values of the five powders were as follows: 115.35, 77.93, 39.38, 25.01, and 20.44 μm, respectively. This demonstrated that ultrafine soybean residue powders were obtained by superfine grinding, and their qualities such as bioavailability, absorption, and solubility would be improved.

**Table 1:** Particle size distributions, micromeritic parameters, color properties, angle of repose, hydration, and oil binding properties of soybean residue powders with different size*

Samples	M60	M100	M200	M300	M400
Particle size distributions					
D_10_	11.29 ± 0.78^a^	10.02 ± 0.12^b^	7.48 ± 0.04^c^	5.69 ± 0.03^d^	5.26 ± 0.01^e^
D_50_	115.35 ± 3.25^a^	77.93 ± 2.31^b^	39.38 ± 0.05^c^	25.01 ± 0.12^d^	20.44 ± 0.07^e^
D_90_	311.33 ± 7.39^a^	196.20 ± 0.88^b^	105.30 ± 1.20^c^	72.02 ± 0.15^d^	61.17 ± 0.30^e^
Span†	2.60 ± 0.02^c^	2.36 ± 0.01^e^	2.48 ± 0.03^d^	2.65 ± 0.01^b^	2.73 ± 0.01^a^
Specific surface area(m^2^/kg)	96.46 ± 4.68^e^	106.42 ± 1.08^d^	144.88 ± 1.48^c^	182.08 ± 1.22^b^	198.32 ± 0.62^a^
Surface‐number mean (μm)	23.07 ± 1.11^a^	20.87 ± 0.21^b^	15.65 ± 0.16^c^	12.20 ± 0.08^d^	11.20 ± 0.03^e^
Volume‐surface mean (μm)	141.70 ± 3.63^a^	92.90 ± 0.46^b^	49.28 ± 0.29^c^	33.03 ± 0.10^d^	27.96 ± 0.11^e^
Angle of repose (°)	48.30 ± 0.84^a^	42.06 ± 1.73^b^	37.87 ± 0.35^c^	34.877 ± 0.32^d^	31.46 ± 1.13^e^
Color properties					
*L*	63.96 ± 0.87^e^	72.00 ± 0.57^d^	77.91 ± 0.17^c^	83.86 ± 0.32^b^	85.95 ± 025^a^
*a*	4.03 ± 0.07^a^	3.13 ± 0.11^b^	2.60 ± 0.08^c^	1.96 ± 0.06^d^	1.14 ± 0.06^e^
*b*	24.20 ± 0.50^a^	19.97 ± 1.95^b^	15.01 ± 0.41^c^	10.28 ± 0.74^d^	8.21 ± 0.24^e^
Water holding capacity (g/g)	7.86 ± 0.46^a^	6.81 ± 0.23^b^	5.27 ± 0.05^c^	4.85 ± 0.10^d^	4.39 ± 0.06^e^
Oil binding capacity(g/g)	1.78 ± 0.10^a^	1.59 ± 0.10^b^	1.58 ± 0.03^b^	1.44 ± 0.03^b^	1.42 ± 0.01^c^
Water solubility index (%)	5.64 ± 0.57^c^	6.99 ± 1.00^bc^	9.31 ± 1.52^a^	8.29 ± 1.40^ab^	6.64 ± 0.57^bc^
Swelling capacity (ml/g)	2.05 ± 0.09^e^	3.64 ± 0.57^d^	7.12 ± 0.33^c^	9.19 ± 0.46^b^	10.62 ± 0.22^a^

* Mean values in the same column with different letters are significantly different (*p* < .05).

^†^ Span was determined by the equation: span = (*d*_(90)_ − *d*_(10)_)/*d*_(50)_.

Table 1 lists the spans of the five soybean residue powders. The “span” is the width of the particle size distribution. A small span value indicated that the powder was homogenous. Compared with the span of M60, the span values first decreased (from 2.60 to 2.48) and then increased (from 2.48 to 2.73) as the size of the soybean residue powder decreased. This behavior was inconsistent with the conclusion that there was a smaller value of span in the superfine powder (Huang, Dou, Li, & Wang, 2018; Zhang, Xu, & Li, 2009), but it disagreed with results for *Dendrobium officinale* (Meng et al., 2017). The differences may have been due to differences in the milling and sieving processes.

As shown in Table 1, with decreasing particle size of the powders, we found that the specific surface area increased from 96.46 to 198.32; the surface‐number mean and volume‐surface mean decreased from 23.07 to 11.20 μm and 141.70 to 27.96 μm. The differences in surface properties were statistically significantly (*p* < .05). These results for soybean agreed with the findings for pomegranate peel and *Dendrobium officinale* (Meng, Fan, Chen, Xiao, & Zhang, 2018; Zhong et al., 2016). Finer powders tended to have a greater number of particles per unit weight, which resulted in their rapid dissolution.

### 3.2 Color

Table 1 presents the colors of the soybean residue powders. The values of *L*, *a*, and *b* varied from 63.96 to 85.95, 4.03 to 1.14, and 24.20 to 8.21, and they showed significant differences between groups (*p* < .05). *L* values increased with decreasing size of the soybean powder, whereas *a* and *b* values decreased only slightly. These results indicated that a small particle size produced relatively brighter and slightly yellow powder. Color is an important quality that affects consumer choice of foods.

### 3.3 Angle of repose

We found that the angles of repose decreased from 48.30° to 31.46° with decreasing particle size (Table 1), and the differences were significant between samples (*p* < .05). These results indicated that the powders with small particle size had better fluidity. The superfine powders did not agglomerate easily because of their fiber and protein content. These effects agreed with findings by Meng et al. (2017), and Ming, Chen, Hong, and Li (2015).

### 3.4 WHC and OBC

As shown in Table 1, as the size of the soybean residue powder decreased, we observed reduction of WHC. The WHCs of M100 (6.81 g/g), M200 (5.27 g/g), M300 (4.85 g/g), and M400 (4.39 g/g) were lower than that of M60 (7.86 g/g). This finding agreed with results of Ming et al. (2015) and Zhang et al. (2009), but they disagreed with the reports of He et al. (2019), Meng et al. (2018), Zhong et al. (2016). The differences may have been caused by hydrophilic groups in the cellulose and hemicelluloses of the soybean residue powders.

The OBCs of the powders decreased when the size of the powder decreased (Table 1). The OBC values ranged from 1.78 to 1.42 g/g. This pattern indicated that superfine grinding had a negative effect on OBC of the soybean residue powders. This effect occurred because the structure of dietary fiber was damaged and its content decreased during processing. Other investigators have observed similar results for the superfine powders of water dropwort and red grape pomace (He et al., 2019; Zhao, Zhu, Zhang, & Tang, 2015).

### 3.5 SC and WSI

We found that SC increased significantly, from 2.05 to 10.62 ml/g, with decreasing particle size (Table 1). Compared with M60 (5.64%), the WSI of M100, M200, M300, and M400 increased by 1.35%, 3.67%, 2.65%, and 1.00%, respectively. The initial increase in WSI followed by a decrease with decreasing particle size could be explained by the fact that the surface area of the powders increased by the superfine grinding, resulting in increasing dispersibility and solubility. However, the soluble carbohydrates and dietary fibers in the superfine powders would be damaged if the milling time was too long. Our results disagreed with He et al. (2019), Jiang et al. (2017), and Zhong et al. (2016).

### 3.6 Antioxidant activity

**Figure 1:**
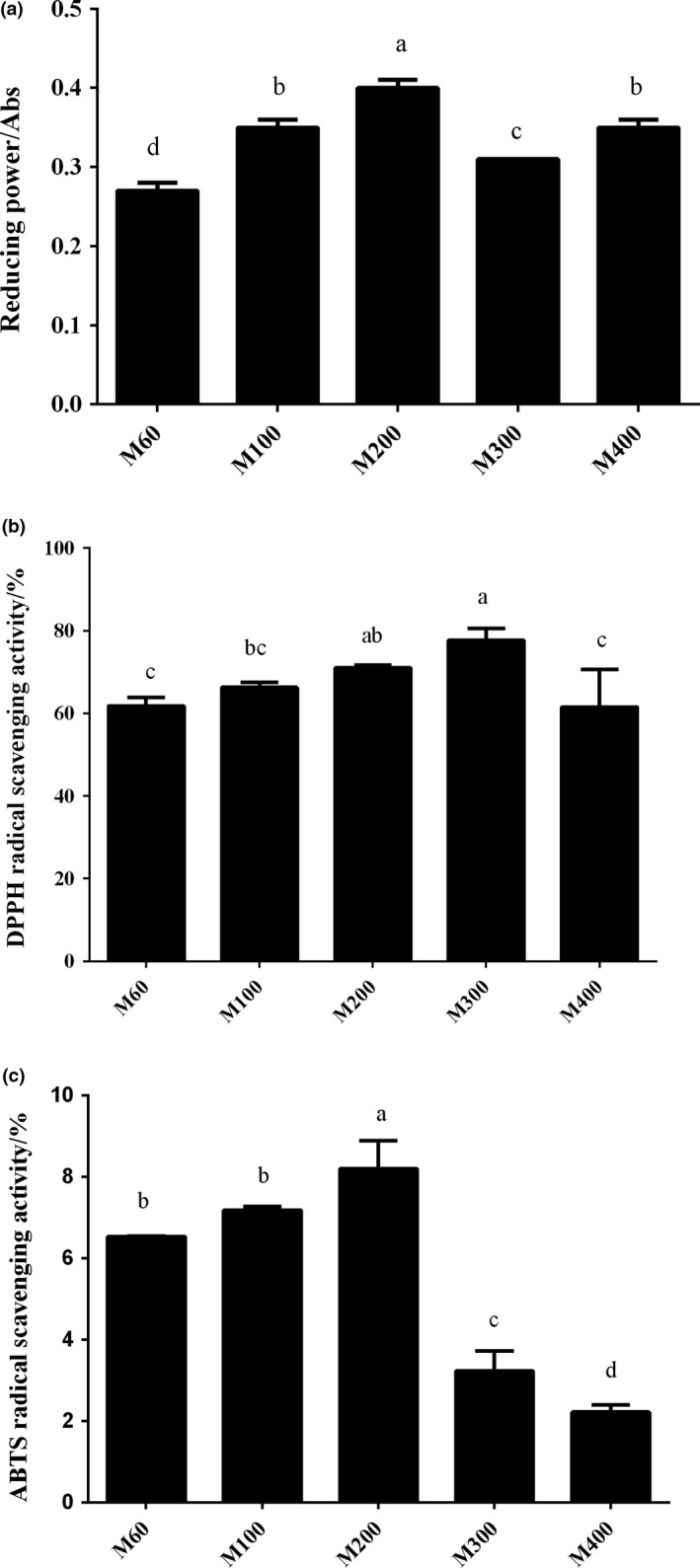
The reducing power (a), DPPH (b) and ABTS (c) radical scavenging activity of the soybean residue powders with different sizes. Shown are the mean and *SD* of three independent experiments. The different letters in the same column are significantly different (*p* < .05)

Figure 1 shows the reducing power, the DPPH and ABTS radical scavenging activities of the soybean residue powders with different particle sizes. We found that reducing power first increased and then decreased with decreasing particle size. The M200 showed the highest reducing power with a value of 0.40, followed by M100, M400, M300, and M60, with values of 0.35, 0.35, 0.31, and 0.27, respectively. The differences in these values were statistically significant (*p* < .05). For the DPPH radical scavenging activity, we observed a similar trend, that is, the values were 61.83%, 66.28%, 71.07%, 77.67%, and 61.54%, for M60, M100, M200, M300, and M400, respectively. We did not find a statistically significant difference in DPPH radical activity between the M200 and M300 (*p* > .05). For the ABTS radical scavenging activity, we found an initial increase and then decrease in soybean dregs powders. The M200 had the highest ABTS radical scavenging rate with a value of 8.19%, followed by M100, M60, M300, and M400 with the values of 7.18%, 6.53%, 3.23%, and 2.22%, respectively. There was a significant difference between the M200 and the other samples (*p* < .05). The results suggested that superfine grinding improved the radical scavenging capacity and reducing power; however, a decline in antioxidant activity would occur if the powder size was too small. Because the release of antioxidant components in soybean residue powder increased as the size of powders decreased, the antioxidant activity of superfine powder increased quickly. However, the antioxidant components could be damaged if milling was too long. Our findings were not in accordance with the results of Chen, Fu, Yue, and Cheng (2015), Zhao et al. (2015), Meng et al. (2017), and Zhong et al. (2016).

## 4 CONCLUSION

We used superfine and conventional grinding methods to produce five kinds of soybean residue powders having different particle sizes. As the particle size decreased, the surface area (from 96.46 to 198.32 m^2^/kg) and swelling capacity (from 2.05 to 10.62 ml/g) increased, and decreases occurred in the surface‐number mean (from 23.07 to 11.20 μm), volume‐surface mean (from 141.70 to 27.96 μm), angle of repose (from 48.30° to 31.46°), water holding capacity (from 7.86 to 4.39 g/g), and oil binding capacity (from 1.78 to 1.42 g/g). The water solubility index and antioxidant activity (reducing power, DPPH and ABTS radical scavenging activity) improved as the size of the particle decreased. Our results demonstrated that superfine grinding could improve some properties of soybean residue powder. Further investigations will assess the effects of superfine grinding on other properties, such as absorption, chemical composition, and morphology of soybean residue micropowders.

## CONFLICT OF INTEREST

The authors declare that they have no conflict of interest.

## ETHICAL APPROVAL

The study did not involve any human or animal testing.
